# Euthyroid Sick Syndrome (ESS) and Monocyte-to-Lymphocyte Ratio (MLR) Are Predictors of Complications in Geriatric Hip Fractures: A Single-Center Retrospective Analysis

**DOI:** 10.3390/jcm15062282

**Published:** 2026-03-17

**Authors:** Giacomo Capece, Doriana Di Costa, Elisa Pesare, Michele Pomponi, Valeria Maccauro, Rocco Maria Comodo, Rami Kaplan, Umberto Capece, Pasquale Farsetti, Marcello Covino, Giulio Maccauro, Raffaele Vitiello

**Affiliations:** 1 Department of Orthopedics and Geriatric Sciences, Catholic University of the Sacred Heart, Largo Francesco Vito 8, 00168 Rome, Italy; doriana.dicosta01@icatt.it (D.D.C.); michele.pomponi01@icatt.it (M.P.); roccocomodo96@gmail.com (R.M.C.); giulio.maccauro@unicatt.it (G.M.); lele.vitiello@gmail.com (R.V.); 2 Department of Orthopedics, Ageing and Rheumatological Sciences, Fondazione Policlinico Universitario Agostino Gemelli IRCCS, Largo Agostino Gemelli 8, 00168 Rome, Italy; 3 Orthopaedic & Trauma Unit, Department of Basic Medical Sciences, Neuroscience and Sense Organs, School of Medicine, AOU Consorziale “Policlinico”, University of Bari Aldo Moro, 70121 Bari, Italy; elisapesare@gmail.com; 4 Department of Internal Medicine and Gastroenterology, Fondazione Policlinico Universitario Agostino Gemelli IRCCS, 00168 Rome, Italy; valeria.maccauro01@icatt.it; 5 Endocrinology and Diabetology Unit, Fondazione Policlinico Universitario Agostino Gemelli IRCCS, 00168 Rome, Italy; capeceumberto@gmail.com; 6 Section of Orthopaedics and Traumatology, Department of Clinical Science and Translational Medicine, University of Rome “Tor Vergata”, 00133 Rome, Italy; farsetti@uniroma2.it; 7 Department of Emergency Unit, Catholic University of the Sacred Heart, Largo Francesco Vito 8, 00168 Rome, Italy; marcello.covino@unicatt.it

**Keywords:** hip fractures, euthyroid sick syndrome, monocyte-to-lymphocyte ratio, geriatrics, inflammatory markers

## Abstract

**Background**: Euthyroid Sick Syndrome (ESS) is a clinical condition characterized by reduced free triiodothyronine (FT3) levels in response to acute or chronic stress. Proximal femur fractures in geriatric patients are associated with high morbidity and mortality rates and ESS may influence outcomes in this population. This study aimed to investigate the role of ESS as a predictor of complications in elderly patients with hip fractures, analyzing its association with inflammatory and nutritional markers, including the Monocyte-to-Lymphocyte Ratio (MLR), Controlling Nutritional Status (CONUT) score and Hemoglobin, Albumin, Lymphocyte, and Platelet (HALP) score. **Materials and Methods**: We conducted a single-center retrospective analysis of patients aged 65 and older who were admitted with proximal femur fractures requiring surgical intervention. Thyroid hormone profiles, inflammatory markers and other clinical variables were analyzed preoperatively (T0) and on the first (T1) and third (T2) postoperative days. Logistic regression was used to identify predictors of complications and transfusion requirements. **Results**: The study included 103 patients (72 men, 31 women; mean age 85 ± 6.27 years). ESS was present in 30 patients (29%) and was associated with longer surgery duration (83.9 ± 35.5 vs. 68.9 ± 21.3 min; *p* = 0.042). At admission (T0), ESS patients had lower FT3 (1.91 ± 0.25 vs. 2.75 ± 0.28 pmol/L; *p* < 0.001) and higher TSH (1.55 ± 0.75 vs. 1.20 ± 0.80 mIU/L; *p* = 0.057). Postoperatively, MLR was significantly altered at T1 (*p* = 0.026) and T2 (*p* = 0.040). ESS was a significant predictor of complications at T0 but lost significance postoperatively, while MLR emerged as a key predictor at T2 (*p* = 0.003). Logistic regression confirmed MLR at T2 as an independent predictor of complications. Hemoglobin levels at T0 (*p* < 0.001), T1 (*p* < 0.001), and T2 (*p* < 0.001), along with albumin at T1 (2.67 ± 0.34 vs. 2.94 ± 0.33 g/dL; *p* = 0.001) and calcium at T1 (*p* = 0.006), were significant predictors of transfusion requirements. Nutritional and inflammatory scores were not predictive. **Conclusions**: ESS is a significant initial predictor of complications in geriatric patients with hip fractures, but inflammatory markers such as MLR assume greater relevance in the postoperative period. These findings emphasize the importance of monitoring ESS and MLR to enhance risk stratification and guide personalized management strategies.

## 1. Introduction

The term Euthyroid Sick Syndrome(ESS) refers to a clinical condition characterized by low levels of free triiodothyronine (FT3) due to impaired conversion of thyroxine (FT4) to triiodothyronine in response to acute or chronic stress [1].

ESS has been documented in various acute and chronic illnesses as a risk factor for increased mortality rates. Recent evidence has highlighted the prognostic implications of ESS for poorer outcomes in hospitalized patients, particularly when associated with comorbid conditions such as hepatopathy, nephropathy, pneumopathy, and tumors [2]. This syndrome typically presents low-to-normal levels of FT4 and normal levels of thyroid-stimulating hormone (TSH). Factors contributing to ESS include reduced protein binding of T4 and diminished cellular uptake of thyroid hormones. Acute clinical events may lead to alterations in the hypothalamic–pituitary–thyroid axis, which in turn impacts the metabolism and transport of thyroid hormones [1].

Notably, immediate declines in mean FT3 concentrations have been observed in orthopedic patients following surgery [3]. Proximal femur fractures are a significant cause of disability and mortality among individuals over the age of 65 and are among the most common fragility fractures [4]. These injuries are typically the result of low-energy trauma, particularly in patients with osteoporosis. Given the aging population, the incidence of these fractures is anticipated to rise. The mortality rate associated with proximal femur fractures is reported to be between 20% and 35%. Key risk factors contributing to a higher mortality risk include advanced age, male sex, socioeconomic deprivation, and the presence of dementia [5].

Recent studies [3] have suggested a possible link between ESS and acute stress responses in geriatric patients with proximal femur fractures, particularly those who exhibit a greater need for red blood cell transfusions compared to control groups. This association correlates with an elevated risk of perioperative anemia, abnormal Vitamin D–PTH axis function, and lower body mass index (BMI) [6]. In addition, inflammatory markers such as the Monocyte-to-Lymphocyte Ratio (MLR) and nutritional scores such as CONUT (Controlling Nutritional Status) and HALP (Hemoglobin, Albumin, Lymphocyte, and Platelet) are emerging as potential predictors of complications and prognosis in surgical patients [7,8,9].

The aim of our research was to evaluate the prognostic role of ESS and other clinical biomarkers, including MLR, CONUT, and HALP scores, in predicting postoperative complications in elderly patients with proximal femur fractures. Identifying early predictors of adverse outcomes may support clinicians in stratifying patient risk and tailoring perioperative management strategies.

## 2. Materials and Methods

### 2.1. Study Design

This study is a retrospective observational analysis conducted at a single medical center. It included all patients aged 65 and older who were admitted to our emergency department between March 2023 and April 2024 with a diagnosis of proximal femur fractures that required surgical intervention. No randomization protocol was applied, as all eligible patients were consecutively included. Data collection was performed retrospectively from electronic medical records and anonymized before analysis to ensure impartiality and reduce bias.

The sample size was not based on a priori statistical calculations but rather included all consecutive patients meeting the eligibility criteria during the study period.

### 2.2. Inclusion and Exclusion Criteria

Patients were eligible for inclusion if they were over 65 years of age, had a proximal femoral fracture confirmed by X-ray and classified as AO type 31A or 31B, required surgical intervention [10], and were admitted to the emergency room within 72 h of the trauma. Exclusion criteria included any known thyroid-related conditions, acute coronary syndrome, active pneumonia, concurrent neoplastic diseases, use of medications known to affect thyroid function (e.g., calcium carbonate, cholestyramine, dopamine agonists, estrogens, growth hormone, octreotide, spironolactone, and sulfonylureas), and refusal to undergo surgery. To ensure exclusion of acute coronary syndrome and active pneumonia at baseline, all patients underwent standardized preoperative evaluation, including electrocardiogram and troponin testing in cases of chest pain or cardiovascular risk, chest X-ray, and inflammatory markers (C-reactive protein and, where clinically indicated, procalcitonin). Only patients without clinical, laboratory, or radiographic evidence of these conditions were included. Postoperative complications therefore represent newly developed events and not pre-existing conditions.

**Figure 1:**
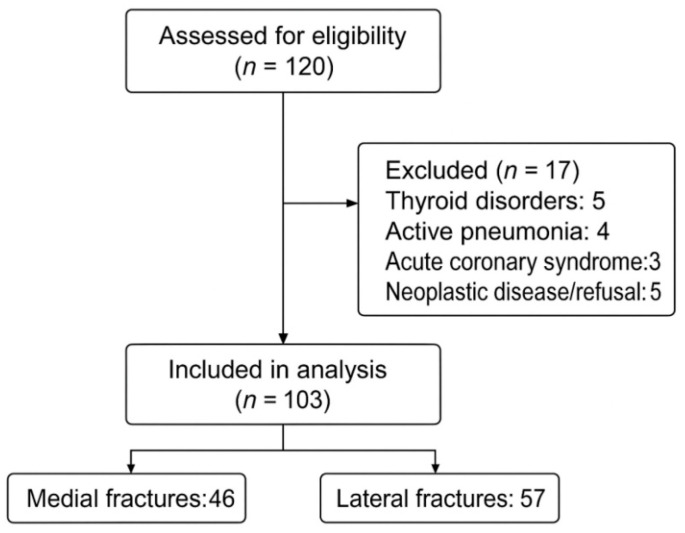
CONSORT flow diagram of participant enrollment and analysis.

Patient selection and exclusion are detailed in Figure 1, illustrating the reasons for exclusion and the final analyzed cohort (n = 103).

### 2.3. Study Variables

Before surgery, all patients had routine blood tests with a comprehensive thyroid hormone profile assessment and attended an initial preoperative evaluation. The same laboratory tests conducted before the operation were repeated on the first and third days following surgery. Laboratory values, including hemoglobin levels, platelet counts, white blood cell counts, fibrinogen levels, and creatinine levels—were recorded along with their trends. Gender, age, fracture type, length of hospital stay, waiting period prior to surgery, and comorbidities, including dementia, diabetes mellitus, obesity, mild to severe liver disease, chronic pulmonary disease, and cardiovascular conditions (including ischemic heart disease, stroke, congestive heart failure, and peripheral artery disease) were among the data gathered upon recovery. Using previous comorbidity data, the Charlson Comorbidity Index was computed.

The surgical decision was made by a qualified orthopedic surgeon based on the fracture type, and a consistent surgical technique was applied to similar types of fractures. Informed consent was waived due to the retrospective and anonymized nature of the study design. Clinical postoperative complications, such as infections at the surgical site, pneumonia, urinary tract infections, myocardial infarctions, fever, sepsis, and postoperative anemia, were documented.

#### Laboratory Assays and Biomarker Calculations

Thyroid hormones (FT3, FT4, TSH) were measured using the Abbott Architect i2000 immunoassay system (Abbott Laboratories, Abbott Park, IL, USA). Reference ranges were: FT3, 2.3–4.2 pmol/L; FT4, 9–20 pmol/L; TSH, 0.4–4.0 mIU/L. Blood samples were collected at standardized time points relative to surgery: T0 (admission, within 72 h of injury), T1 (first postoperative day), and T2 (third postoperative day). Samples were processed according to the manufacturer’s instructions.

Nutritional and inflammatory scores were computed as follows: CONUT score was calculated based on serum albumin, total lymphocyte count, and total cholesterol. Scores range from 0 to 12, with higher scores indicating worse nutritional status. HALP score was calculated using the formula: HALP = (Hemoglobin [g/L] × Albumin [g/L] × Lymphocytes [10^9^/L]) ÷ Platelets [10^9^/L]. All calculations were performed using raw laboratory values at each time point. Cutoffs for ESS classification and other risk stratifications are detailed in the Section 3.

### 2.4. Study Endpoints

The primary study endpoint was to evaluate the association between euthyroid sick syndrome (ESS) and postoperative complications in geriatric patients with proximal femur fractures. Complications were defined as medical or surgical adverse events during hospitalization, including infections, cardiovascular events, and electrolyte disturbances. Blood transfusions were analyzed separately as a secondary endpoint.

The secondary endpoint was to identify prognostic factors among clinical and laboratory parameters, with a particular focus on inflammatory and nutritional markers such as MLR, CONUT, and HALP, as well as thyroid function tests.

### 2.5. Statistical Analysis

Continuous variables were assessed for normality using the Shapiro–Wilk test. Normally distributed variables are presented as means ± standard deviation (SD), while non-normally distributed variables are reported as medians (interquartile range, IQR). Categorical variables are expressed as counts and percentages.

Missing laboratory data were handled using standard multiple imputation implemented in R (version X.X) with the “mice” package, under the assumption that data were missing at random (MAR). Patients with more than two missing laboratory values were excluded from imputation. Sensitivity analyses were performed to assess the robustness of the imputation.

Univariate analyses were first conducted to identify potential predictors of postoperative complications and transfusion requirements. Continuous variables were compared using *t*-tests (or Mann–Whitney U tests for non-normal distributions), and categorical variables using the Chi-square or Fisher’s exact test as appropriate.

Variables with *p* < 0.05 in univariate analysis were entered into multivariable logistic regression models. The regression outputs are now presented as odds ratios (OR) with 95% confidence intervals (CI) and associated *p*-values for clarity. Multicollinearity was assessed using the variance inflation factor (VIF), and all included variables had VIF < 2. Model calibration was evaluated using the Hosmer–Lemeshow test, and discriminatory performance was assessed with the area under the receiver operating characteristic curve (AUC).

All statistical analyses were performed using R (version X.X), and a two-sided *p*-value < 0.05 was considered statistically significant.

### 2.6. Ethical Approval

The study was conducted in accordance with the principles expressed in the Declaration of Helsinki and its later amendments. The research protocol was approved by the Institutional Review Board of our hospital.

## 3. Results

A total of 120 patients aged 65 years and older with proximal femur fractures were initially assessed for eligibility. Seventeen patients were excluded for the following reasons: thyroid disorders (n = 5), active pneumonia (n = 4), acute coronary syndrome (n = 3) and neoplastic disease or refusal to undergo surgery (n = 5). The final analyzed cohort consisted of 103 patients (Figure 1), including 72 men and 31 women, with a mean age of 85 ± 6.27 years (range: 71–99) (Table 1).

General patient characteristics included a mean BMI of 24 ± 3.72 kg/m^2^ (Table 1). The degree of pre-existing osteoarthritis, according to the Kellgren–Lawrence grade, was as follows: 4 patients at grade 0.33 at grade 1.26 at grade 2.30 at grade 3, and 10 at grade 4 (Table 1). Levels of Activities of Daily Living (ADL) had an average of 4.9 ± 1.97 (range: 0–6), while Instrumental Activities of Daily Living (IADL) averaged 4.9 ± 3 (range: 0–8) (Table 1). Fractures were classified according to the AO classification for proximal femoral fractures (31). Among these, 10 fractures were classified as A1.2, 12 as A1.3, 13 as A2.2, 7 as A2.3, 4 as A3.3, 4 as B1.1, 9 as B1.2, 11 as B1.3, 19 (the most common) as B2.1, 2 as B2.2, 6 as B2.3, and 6 as B3. These fractures were further grouped into medial fractures (46 cases) and lateral fractures (57 cases) (Table 1). The mean time from diagnosis to surgery was 31 ± 19.96 h (range: 4–62 h), and the mean duration of surgery was 78 ± 32 min (range: 34–201 min) (Table 1).

Three surgical techniques were employed: cannulated screws in 11 cases, intramedullary nails in 56 cases, and prostheses in 36 cases (Table 1). The anesthesia methods utilized included local anesthesia in 7 cases, spinal anesthesia in 50 cases, and general anesthesia in 46 cases. Postoperatively, 39 patients required blood transfusions (37.8%) (Table 1).

Regarding postoperative complications, 13 patients (12.6%) experienced pulmonary complications, including pneumonia (diagnosed via chest X-ray and clinical assessment of respiratory symptoms) and respiratory insufficiency requiring oxygen therapy. Urinary complications occurred in 15 patients (14.6%), including urinary tract infections (diagnosed based on urinalysis and urine culture) and acute urinary retention requiring catheterization. Wound-related complications were observed in 2 patients (1.9%), including hematoma and delayed wound healing, diagnosed by clinical examination. Other complications (6 cases, 5.8%) included deep vein thrombosis (diagnosed by Doppler ultrasound) and electrolyte imbalances requiring medical intervention. There were no recorded cases of surgical site infections or implant failures. Notably, 67 patients (65%) experienced no complications (Table 1).

**Figure 2:**
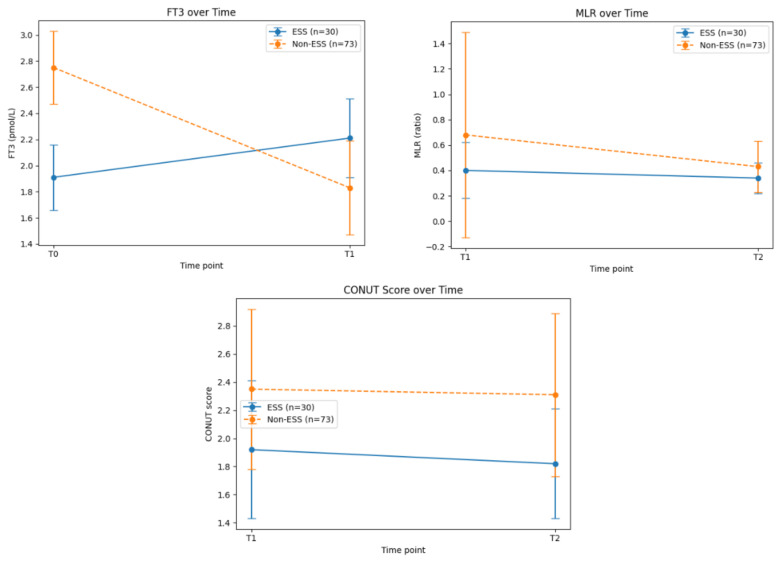
FT3 (pmol/L), MLR (lymphocyte-to-monocyte ratio), and CONUT score are shown at T0 (admission), T1 (1st postoperative day), and T2 (3rd postoperative day) in patients with and without Euthyroid Sick Syndrome (ESS). ESS patients are indicated by solid lines with filled markers, non-ESS patients by dashed lines with open markers. FT3 levels normalize postoperatively in ESS patients, while MLR and CONUT show persistent alterations, with MLR at T2 significantly predicting postoperative complications.

Among all patients enrolled, 30 (29%) were classified as having Euthyroid Sick Syndrome (ESS), defined retrospectively as FT3 < 2.4 pmol/L at admission (T0), a cut-off previously used in comparable geriatric and surgical cohorts. In accordance with this definition, mean FT3 levels at T0 were significantly lower in the ESS group compared to the non-ESS group (1.91 ± 0.25 vs. 2.75 ± 0.28 pmol/L; *p* < 0.001). This simplified criterion was chosen because the retrospective dataset did not allow complete assessment of FT4 and TSH in all patients (Table 2) (Figure 2). Analysis of patients with ESS revealed several significant alterations associated with this condition. Specifically, the duration of surgery was significantly longer in the ESS subgroup (83.90 ± 35.50 min vs. 68.90 ± 21.31 min in non-ESS; *p* = 0.042) (Table 2). At T0, the following average levels were altered: TSH 1.55 ± 0.75 mIU/L (vs. 1.20 ± 0.80; *p* = 0.057), PTH 70.39 ± 36.97 pg/mL (vs. 94.02 ± 45.54; *p* = 0.020), and Vitamin D 23.64 ± 13.24 ng/mL (vs. 18.76 ± 7.48; *p* = 0.043). During follow-up at T1 (first postoperative day), alterations were observed in lymphocytes (2.82 ± 3.86 ×10^9^/L vs. 1.11 ± 0.41; *p* = 0.004), MLR (0.40 ± 0.22 vs. 0.68 ± 0.81; *p* = 0.026), calcium (8.77 ± 0.54 mg/dL vs. 8.49 ± 0.51; *p* = 0.029), FT3 (2.21 ± 0.30 pmol/L vs. 1.83 ± 0.36; *p* < 0.001), CONUT score (1.92 ± 0.49 vs. 2.35 ± 0.57; *p* = 0.043), and HALP score (4.28 ± 6.53 vs. 1.66 ± 0.80; *p* = 0.008). At T2 (third postoperative day), MLR remained lower in ESS patients (0.34 ± 0.12 vs. 0.43 ± 0.20; *p* = 0.040), while CONUT score differences persisted (1.82 ± 0.39 vs. 2.31 ± 0.58; *p* = 0.001) (Table 2) (Figure 2). These findings suggest a sustained postoperative alteration in lymphocyte–monocyte balance in ESS patients.

The analysis of postoperative complications showed that patients with complications had a lower FT4 level at admission (T0), with a mean value of 11.11 ± 2.35 pmol/L (vs. 12.53 ± 2.12 pmol/L; *p* = 0.014) (Table 3).

FT3 was a significant predictor at T1, with a mean value of 1.83 ± 0.47 pmol/L (vs. 2.03 ± 0.34 pmol/L; *p* = 0.043), but not at T2 (1.83 ± 0.52 vs. 2.86 ± 3.82 pmol/L; *p* = 0.328) (Table 3). In the advanced postoperative period (T2), MLR emerged as the primary predictor of complications, with a significant *p* = 0.003 (0.49 ± 0.23 vs. 0.36 ± 0.14) (Table 3). ESS was a strong predictor at admission but lost statistical significance postoperatively, likely due to normalization of FT3 levels caused by surgical stress and hormonal responses. Among inflammatory markers, the Systemic Inflammatory Response Index (SIRI) did not reach statistical significance.

Analysis revealed that significant predictors of transfusion requirements included hemoglobin (Hb) levels at T0 (11.20 ± 1.74 g/dL vs. 12.77 ± 1.44 g/dL; *p* < 0.001), T1 (8.85 ± 1.19 g/dL vs. 10.73 ± 1.28 g/dL; *p* < 0.001), and T2 (8.84 ± 0.91 g/dL vs. 9.81 ± 1.10 g/dL; *p* < 0.001), albumin levels at T1 (2.67 ± 0.34 g/dL vs. 2.94 ± 0.33 g/dL; *p* = 0.001) and at T2 (2.56 ± 0.28 g/dL vs. 2.78 ± 0.35 g/dL; *p* = 0.006), calcium levels at T1 (8.83 ± 0.63 mg/dL vs. 8.72 ± 0.43 mg/dL; *p* = 0.006) and T2 (8.38 ± 0.46 mg/dL vs. 8.62 ± 0.60 mg/dL; *p* = 0.077), and white blood cell (WBC) count at T2 (8.83 ± 2.67 × 10^9^/L vs. 7.53 ± 2.37 × 10^9^/L; *p* = 0.030). Nutritional and inflammatory scores, including CONUT and HALP, were not useful predictors of transfusion risk (Table 4).

A logistic regression analysis was performed to identify predictors of complications using age, FT3, MLR at T0, T1, and T2, and HALP at T0 and T2 as independent variables (Table 5).

MLR emerged as the most significant predictor during the advanced postoperative period (T2), with a *p*-value of 0.003 [Table 5]. HALP and other variables included in the regression model did not reach statistical significance [Table 5] (Figure 2). The large odds ratio observed for MLR at T2 (OR = 18.7, 95% CI 1.9–182.4, *p* = 0.003) should be interpreted with caution. This high value likely reflects the strong association between postoperative systemic inflammation and complications in our cohort, while also being influenced by the limited sample size and potential residual confounding factors. Sensitivity analyses using alternative modeling strategies confirmed the robustness of the association, although the magnitude of the OR may vary.

## 4. Discussion

The relationship between acute stress responses and thyroid dysfunction in patients with proximal femur fractures raises important questions about the impact of ESS, as evidenced by our findings. In this study, we explored the role of ESS and the MLR as potential predictors of postoperative complications in geriatric patients with proximal femur fractures.

In the advanced postoperative period (T2), MLR emerged as the primary predictor of complications, with a significant *p*-value of 0.003 (0.49 ± 0.23 vs. 0.36 ± 0.14) (Table 3). Our results underscore the importance of ESS as an early predictor of complications upon admission, with inflammatory markers, particularly MLR, gaining greater significance in the postoperative period [9]. Given the limited number of patients experiencing complications, caution is warranted when interpreting effect estimates for predictors at multiple time points. The large odds ratio observed for MLR at T2 likely reflects the strong association with postoperative complications in our cohort, but the magnitude may be inflated due to the small sample size and residual confounding. Therefore, these results should be viewed as indicative rather than definitive, and further studies with larger cohorts are needed to confirm these findings.

ESS is a well-established phenomenon in critically ill patients and has been linked to various acute and chronic conditions, including trauma and fractures [11]. According to our evidence, patients with complications had a lower FT4 level at admission (T0), with a mean value of 11.11 ± 2.35 pmol/L (vs. 12.53 ± 2.12 pmol/L; *p* = 0.014) and FT3 mean value of 1.83 ± 0.47 pmol/L (2.03 ± 0.34 pmol/L; *p* = 0.043) at T1 was a predictor of complication, but not at T2 (1.83 ± 0.52 pmol/L vs. 2.86 ± 3.82 pmol/L; *p* = 0.328) (Table 3). Notably, even alterations in thyroid hormone levels occurred, particularly in low FT3 (Table 2). This hormonal imbalance has consistently been associated with poor outcomes in several conditions, including hip fractures, further supporting the idea that thyroid hormone dysregulation serves as an early marker of stress response in severely ill patients [12,13]. However, as the postoperative period progressed, ESS lost its statistical significance at T2, likely due to the normalization of thyroid hormone levels following surgical stress or hormonal adaptations. Previous research suggests that the hormonal changes seen in ESS may be transient, potentially reversing once the acute stressor, such as surgery, resolves [14].

While ESS lost its predictive value for postoperative complications, MLR emerged as a more reliable predictor at T2, with a significant *p*-value of 0.003 (0.49 ± 0.23 vs. 0.36 ± 0.14). This is consistent with recent studies identifying MLR as a strong marker of systemic inflammation and poor outcomes in surgical patients [15]. MLR represents the balance between monocytes, which mediate inflammation, and lymphocytes, crucial for immune responses [16]. An elevated MLR has been linked to increased risk of complications, such as infections, delayed wound healing, and cardiovascular events in various patient populations, including geriatric fracture patients [17,18]. Our study further supports the growing body of evidence suggesting that MLR could be an important tool for assessing postoperative risk in geriatric hip fracture patients. Given its ease of calculation and availability through routine blood tests, MLR could be integrated into clinical practice as part of a comprehensive risk stratification approach [19] (Table 2). The high odds ratio observed for MLR at T2 highlights its strong association with postoperative complications. This result should be interpreted cautiously, as the magnitude may be influenced by the relatively small sample size and residual confounding. Biologically, it reflects the key role of systemic inflammation in the early postoperative period, with monocyte–lymphocyte imbalance potentially promoting infection, delayed wound healing, and other complications. Sensitivity analyses applying alternative logistic regression models confirmed the persistence of the association, supporting the robustness of MLR as a postoperative prognostic marker.

In contrast to MLR, other inflammatory and nutritional scores, such as CONUT and HALP, were not found to be effective predictors of complications or transfusion requirements in our cohort: CONUT score 1.92 ± 0.49 (2.35 ± 0.57; *p* = 0.043), and HALP score 4.28 ± 6.53 (1.66 ± 0.80; *p* = 0.008). These markers, while associated with outcomes in other surgical populations [20,21,22], did not provide additional predictive value for geriatric hip fracture patients undergoing surgery. Similar observations have been made in prior studies where traditional nutritional and inflammatory scores were less sensitive or specific for predicting outcomes in this patient group. The failure of CONUT and HALP to predict complications in our study could be attributed to the multifactorial nature of postoperative outcomes in geriatric patients, which involve not only inflammatory and nutritional status but also frailty, comorbidities, and functional impairment. Therefore, these scores may need to be supplemented with other biomarkers or clinical assessments in the context of geriatric hip fractures. For the detailed CONUT and HALP scores (Table 3).

Our analysis also identified several hematological and biochemical parameters, including Hb, albumin, and calcium. Hb levels at T0, T1 and T2 were all significant with *p* < 0.001: T0 (11.20 ± 1.74 g/dL vs. 12.77 ± 1.44 g/dL; *p* < 0.001), T1 (8.85 ± 1.19 g/dL vs. 10.73 ± 1.28 g/dL; *p* < 0.001), and T2 (8.84 ± 0.91 g/dL vs. 9.81 ± 1.10 g/dL; *p* < 0.001). Furthermore albumin levels at T1 (2.67 ± 3.44 g/dL vs. 2.94 ± 3.36 g/dL; *p* = 0.001) and T2 (2.56 ± 2.82 g/dL vs. 2.78 ± 3.48 g/dL; *p* = 0.006). Additionally, calcium levels at T1 (8.83 ± 0.63 mg/dL vs. 8.72 ± 0.43 mg/dL; *p* = 0.006) and T2 (8.38 ± 0.46 mg/dL vs. 8.62 ± 0.60 mg/dL; *p* = 0.077) can be considered to be significant predictors of transfusion requirements. These findings align with previous research highlighting the importance of early detection of anemia and malnutrition in patients undergoing orthopedic surgery [23]. The role of albumin and calcium as predictors of complications further underscores the necessity of preoperative optimization, including correcting nutritional deficiencies and metabolic imbalances [24,25]. It is important to note that, unlike hemoglobin, neither MLR nor nutritional scores (CONUT and HALP) were predictive of transfusion requirements in our cohort.

This apparent discrepancy does not necessarily imply that ESS/MLR have no role in the pathogenesis of anemia, as previously reported, but rather suggests that perioperative transfusion risk in geriatric hip fracture patients is mainly driven by surgical blood loss and baseline hemoglobin levels, which outweigh the influence of systemic inflammation or nutritional status. In contrast, MLR retained predictive value for postoperative complications, indicating that its role may be more closely linked to infection and inflammatory processes than to anemia itself. This distinction emphasizes the need to consider different biological mechanisms when evaluating predictors of transfusion versus predictors of broader postoperative complications.

From a clinical perspective, our study emphasizes the value of monitoring ESS upon admission in geriatric patients with hip fractures. While ESS may not predict long-term outcomes, its presence alerts clinicians to the need for careful postoperative monitoring, particularly concerning thyroid function and inflammatory markers [26,27,28]. While transfusion requirements reflect perioperative anemia and baseline hemoglobin, they were not included among postoperative complications, which were limited to infections, cardiovascular events, and electrolyte disturbances.

Additionally, the identification of MLR as a key predictor of complications suggests its potential utility in postoperative care. An elevated MLR could prompt early intervention to mitigate the risk of infections or other inflammatory complications [29,30,31].

### Study Limitations

Despite promising results, our study has several limitations. First, the sample size is relatively small, which may have limited the statistical power to detect subtle differences in predictive markers. Moreover, no formal sample size calculation was performed because of the retrospective nature of the study, which may have further limited statistical power. Larger multicenter studies with more extensive cohorts are necessary to validate our findings and assess their generalizability.

Second, the observational nature of the study limits the ability to draw causal conclusions between ESS, MLR, and postoperative complications. Prospective and longitudinal studies would be beneficial to determine the causal relationship between these markers and outcomes, and to examine how these markers evolve over time.

Third, while we focused on thyroid dysfunction and inflammatory markers, other factors such as frailty, comorbidities, cognitive function, time to surgery, fracture pattern, type of surgery, anesthesia, and perioperative management may influence postoperative outcomes but were not included in the logistic regression models due to the limited sample size and the relatively small number of complication events. These variables represent potential residual confounders, and future studies with larger, multicenter cohorts should evaluate their impact to provide a more comprehensive risk assessment.

Fourth, ESS diagnosis was based solely on FT3 < 2.4 pmol/L. While more comprehensive definitions including FT4 and TSH have been proposed (e.g., Girvent et al.) [11], we opted for this simplified criterion due to incomplete availability of these parameters in our retrospective dataset. Sensitivity analyses applying alternative definitions of ESS incorporating FT4 and TSH confirmed that the main associations with postoperative complications remained consistent. This methodological choice may have contributed to the higher prevalence observed (29%) compared to the 15–25% typically reported in the literature.

Fifth, given the multiple biomarkers assessed across several timepoints and subgroup comparisons, the risk of type I error is increased. No formal correction for multiple testing or pre-specified analysis hierarchy was applied, and statistically significant findings should therefore be interpreted with caution.

Finally, the single-center design, in-hospital-only outcomes, absence of external validation, and lack of post-discharge follow-up limit the generalizability and clinical applicability of our findings. Any implications for risk stratification or management should be considered preliminary.

## 5. Conclusions

This study highlights the prognostic significance of ESS and MLR in geriatric patients undergoing surgery for proximal femur fractures. ESS serves as a valuable early predictor of postoperative complications, particularly at admission, reflecting the acute stress response in this vulnerable population. However, as the postoperative period progresses, inflammatory markers, especially MLR, emerge as stronger and more reliable predictors of complications.

These findings highlight associations between ESS, MLR, and in-hospital complications in geriatric patients with hip fractures. Given the single-center design, absence of post-discharge follow-up, and lack of external validation, conclusions regarding clinical risk stratification and management should be considered preliminary. Further multicenter studies with extended follow-up are needed to validate these findings and explore potential clinical applications.

## Figures and Tables

**Table 1:** Summary of Patient Characteristics, Fracture Classification, and Outcomes in the Retrospective Analysis (BMI = Body Mass Index; ADL = Activities of Daily Living; IADL = Instrumental Activities of Daily Living; AO = AO Foundation).

Category	Details
Number of patients	103
Gender	Men 72 [69.9%], Women 31 [30.1%]
Mean age	85 ± 6.3 years
Mean BMI	24 ± 3.5 kg/m^2^
Degree of osteoarthritis (Kellgren–Lawrence)	Grade 0: 4 [3.9%]; Grade 1: 33 [32%]; Grade 2: 26 [25.2%]; Grade 3: 30 [29.1%]; Grade 4: 10 [9.7%]
Mean ADL score	4.9 ± SD
Mean IADL score	4.9 ± SD
Fracture classification (AO)	A1.2: 10 [9.7%]; A1.3: 12 [11.7%]; A2.2: 13 [12.62%]; A2.3: 7 [6.80%]; A3.3: 4 [3.88%]; B1.1: 4 [3.88%]; B1.2: 9 [8.74%]; B1.3: 11 [10.68%]; B2.1: 19 [18.45%]; B2.2: 2 [1.94%]; B2.3: 6 [5.83%]; B3: 6 [5.83%]
Fracture types	Medial 46 [44.7%]; Lateral 57 [55.3%]
Mean time from diagnosis to surgery	31 ± SD hours
Mean duration of surgery	78 ± SD minutes
Surgical techniques	Cannulated screws 11 [10.7%]; Intramedullary nails 56 [54.4%]; Prostheses 36 [35%]
Type of anesthesia	Local 7 [6.8%]; Spinal 50 [48.5%]; General 46 [44.7%]
Postoperative blood transfusions	39 [37.9%]
Postoperative complications	Pulmonary 13 [12.6%]; Urinary 15 [14.6%]; Wound-related 2 [1.9%]; Others 6 [5.8%]; None 67 [65%]

**Table 2:** Clinical and Biochemical Characteristics of Patients with Euthyroid Sick Syndrome (ESS); T0: Admission to emergency department, T1: First postoperative day, T2:Third postoperative day, n/a: not applicable.

Variable	ESS Group (n = 30)	Non-ESS Group (n = 73)	*p*-Value
Age (years)	85 ± 6.27	85 ± 6.27	n/a
Surgery duration (minutes)	83.90 ± 35.50	68.90 ± 21.31	0.042
FT3 T0 (pmol/L)	1.91 ± 0.25	2.75 ± 0.28	<0.001
TSH T0 (mIU/L)	1.55 ± 0.75	1.20 ± 0.80	0.057
PTH T0 (pg/mL)	70.39 ± 36.97	94.02 ± 45.54	0.020
Vitamin D T0 (ng/mL)	23.64 ± 13.24	18.76 ± 7.48	0.043
Lymphocytes T1 (×10^9^/L)	2.82 ± 3.86	1.11 ± 0.41	0.004
MLR T1	0.40 ± 0.22	0.68 ± 0.81	0.026
Calcium T1 (mg/dL)	8.77 ± 0.54	8.49 ± 0.51	0.029
FT3 T1 (pmol/L)	2.21 ± 0.30	1.83 ± 0.36	<0.001
CONUT score T1	1.92 ± 0.49	2.35 ± 0.57	0.043
HALP score T1	4.28 ± 6.53	1.66 ± 0.80	0.008
MLR T2	0.34 ± 0.12	0.43 ± 0.20	0.040
CONUT score T2	1.82 ± 0.39	2.31 ± 0.58	0.001

**Table 3:** Predictors of Postoperative Complications: logistic regression analyses were adjusted for age and sex. Multicollinearity was assessed using the variance inflation factor (VIF), with all variables showing VIF < 2. Model calibration was evaluated with the Hosmer–Lemeshow goodness-of-fit test (*p* > 0.05), and model discrimination was assessed using the area under the receiver operating characteristic curve (AUC = 0.81), indicating good predictive performance. OR = Odds Ratio; β = adjusted regression coefficient; CI = 95% confidence interval. Significant values are indicated by *p*-values < 0.05. Sensitivity analyses using alternative models (e.g., multiple linear regression) confirmed the robustness of the results. n/a: not applicable.

Group	N° Patients	FT3 T0 (pmol/L)	FT3 T1	FT3 T2	FT4 T0	FT4 T1	FT4 T2	MLR T0	MLR T1	MLR T2
Complications	30	2.04	1.83	1.83	11.11	n/a	n/a	0.58	0.57	0.49
No Complications	73	2.30	2.03	2.86	12.53	n/a	n/a	0.53	0.57	0.36
Adjusted β (age, sex)	n/a	−0.21	−0.18	0.12	−1.35	n/a	n/a	0.05	−0.02	−0.13
95% CI	n/a	−0.39, −0.03	−0.35, −0.01	−0.24, 0.48	−2.42, −0.28	n/a	n/a	−0.10, 0.20	−0.14, 0.10	−0.21, −0.05
*p*-value	n/a	0.022	0.037	0.329	0.014	n/a	n/a	0.524	0.765	0.003
OR		0.81	0.84	1.13	0.78	n/a	n/a	1.05	0.98	18.7

**Table 4:** Predictors of transfusion requirements.

	N. Patients	Hb (g/dL)	Hb (g/dL)	Hb (g/dL)	Albumin (g/dL)	Albumin (g/dL)	Calcium (mg/dL)	Calcium (mg/dL)	Calcium (mg/dL)	wbc (×10^9^/L)	wbc (×10^9^/L)	wbc (×10^9^/L)
Evaluation Time		T0	T1	T2	T1	T2	T0	T1	T2	T0	T1	T2
ESS Group	30	11.20	8.85	8.84	2.67 ± 0.34	2.56 ± 0.28	11.76	8.38	8.38	10.18	9.87	8.83
No ESS Group	73	12.77	10.73	9.81	2.94 ± 0.33	2.78 ± 0.35	9.5	8.72	8.62	10.25	8.95	7.53
*p* Value		<0.001	<0.001	<0.001	0.001	0.006	0.251	0.006	0.077	0.933	0.207	0.03

**Table 5:** Logistic regression analysis: models were adjusted for age and sex. Multicollinearity was assessed using VIF (<2 for all variables). Model calibration was tested with the Hosmer–Lemeshow goodness-of-fit test (*p* > 0.05), and discrimination was evaluated using AUC = 0.84. Sensitivity analyses using alternative models confirmed the robustness of results.

Variable	Coefficient	Standard Error	*p*-Value	Odds Ratio	95% Confidence Interval
Age	−0.0344	0.0551	0.5323	0.9662	(0.8673, 1.0763)
FT3	−1.6557	0.7897	0.0360	0.1910	(0.0406, 0.8977)
MLR 0	−0.6095	0.5992	0.3090	0.5436	(0.1680, 1.7592)
MLR 1	−0.0594	0.4919	0.9039	0.9423	(0.3593, 2.4713)
MLR 3	5.7607	1.9499	0.0031	317.5598	(6.9507, 14,508.4662)
HALP 1	0.1708	0.1088	0.1166	1.1862	(0.9584, 1.4683)
HALP 3	0.0790	0.2019	0.6956	1.0822	(0.7285, 1.6077)
Constant	2.6212	4.9463	0.5962		

## Data Availability

All the data we analyzed and tables we compiled are available for any clarification.
